# A multiplex assay for the sensitive detection and quantification of male and female *Plasmodium falciparum* gametocytes

**DOI:** 10.1186/s12936-018-2584-y

**Published:** 2018-11-29

**Authors:** Lisette Meerstein-Kessel, Chiara Andolina, Elvira Carrio, Almahamoudou Mahamar, Patrick Sawa, Halimatou Diawara, Marga van de Vegte-Bolmer, Will Stone, Katharine A. Collins, Petra Schneider, Alassane Dicko, Chris Drakeley, Ingrid Felger, Till Voss, Kjerstin Lanke, Teun Bousema

**Affiliations:** 1 Department of Medical Microbiology, Radboud University Medical Center, Nijmegen, The Netherlands; 2 Centre for Molecular and Biomolecular Informatics, Radboud Institute for Molecular Life Sciences, Radboud University Medical Center, Nijmegen, The Netherlands; 3 Swiss Tropical and Public Health Institute, Basel, Switzerland; 4 University of Basel, Basel, Switzerland; 5 Malaria Research and Training Centre, University of Science, Techniques and Technologies of Bamako, Bamako, Mali; 6 Human Health Division, International Centre for Insect Physiology and Ecology, Mbita Point, Kenya; 7 Department of Immunology and Infection, Faculty of Infectious and Tropical Diseases, London School of Hygiene and Tropical Medicine, London, UK; 8 Institute of Evolutionary Biology and Institute of Immunology and Infection Research, School of Biological Sciences, University of Edinburgh, Edinburgh, UK

## Abstract

**Background:**

The transmission of malaria to mosquitoes depends on the presence of gametocytes that circulate in the peripheral blood of infected human hosts. Sensitive estimates of the densities of female gametocytes (FG) and male gametocytes (MG) may allow the prediction of infectivity to mosquitoes and thus a molecular estimate of the human infectious reservoir for transmission.

**Methods:**

A novel multiplex qRT-PCR assay with intron-spanning primers was developed for the parallel quantification of FG and MG. *CCp4* (PF3D7_0903800) transcripts specific for FG and *PfMGET* (PF3D7_1469900) transcripts specific for MG were quantified in total nucleic acids. The assay was validated on sex-sorted gametocytes from culture material and on samples from clinical trials with gametocytocidal drugs. Synthetic RNA standards were generated for the two targets genes and calibrated against known gametocyte quantities.

**Results:**

The limit of detection was determined at 0.1 male and 0.1 female gametocyte/µL, which was equal to the limit of quantification (LOQ) for MG, while the LOQ for FG was 1 FG/µL. Results from previously reported clinical trials that used separate gametocyte qRT-PCR assays for FG (targeting *Pfs25*) and MG (targeting *PfMGET*) were reproduced with the multiplex assay. High levels of agreement between separate assays and the multiplex approach were observed (R^2^ = 0.9473, 95% CI 0.9314–0.9632, for FG measured by transcript levels of *Pfs25* in qRT-PCR or *CCp4* in multiplex; R^2^ = 0.8869, 95% CI 0.8541–0.9197, for MG measured by *PfMGET* in either single or multiplex qRT-PCR). FG and MG transcripts were detected in pure ring stage parasites at 10,000- and 100,000-fold reduced frequency for *CCp4* and *PfMGET*, respectively. The *CCp4* and *PfMGET* transcripts were equally stable under suboptimal storage conditions.

**Conclusions:**

Gametocyte densities and their sex ratios can be determined in the presented one-step multiplex assay with higher throughput than single assays. The interpretation of low gametocyte densities at asexual parasite densities above 1000 parasites/µL requires caution to avoid false positive gametocyte signals from spurious transcript levels in ring stage parasites.

**Electronic supplementary material:**

The online version of this article (10.1186/s12936-018-2584-y) contains supplementary material, which is available to authorized users.

## Background

The significant reduction in malaria mortality and morbidity seen in the last 10 years is the result of combined efficient control measures such as early diagnosis, effective treatment with artemisinin-based combination therapy (ACT), active surveillance and vector control. Nevertheless, in 2016, 216 million cases were still reported worldwide [1] and major concerns exist about the extent to which the emergence and spread of insecticide [2] and artemisinin resistance [3] may affect worldwide malaria control and elimination efforts. New strategies may be needed to sustain recent gains and accelerate malaria elimination initiatives. These new strategies include the development and deployment of transmission-blocking strategies that aim to reduce malaria incidence by targeting the infection reservoir involved in maintaining parasite transmission from humans to *Anopheles* mosquitoes.

Transmission to mosquitoes is mediated by the presence of gametocytes in peripheral blood of a human host. Gametocytes are sexually dimorphic and both sexes are required to ensure the development of the parasite inside the mosquito. Although the likelihood of mosquito infection is largely dictated by gametocyte density [4], the gametocyte sex ratio may also play a significant role in ensuring fertilization [5–7]. Since one female gametocyte (FG) produces only one gamete, while a male gametocyte (MG) produces eight gametes [8, 9], the gametocyte sex ratio is usually female-biased in the proportion of 3–5 females: 1 male [10, 11] with indications from rodent malarias and natural *Plasmodium falciparum* infections that sex ratios may be adjusted in the presence of other parasite clones [6, 12], in relation to gametocyte density [7, 13] during infections and in response to environmental factors, such as anaemia [10]. Recent findings further suggest that anti-malarial drugs may have differential effects on MG and FG [14, 15]. Understanding gametocyte sex ratios is thus of interest to understand *Plasmodium* biology, better predict transmission potential during natural malaria infections and estimate the likelihood of onward transmission to mosquitoes after treatment with anti-malarial drugs.

Gametocytes usually circulate in blood at low levels as only 0.2–1% of asexual parasites commit to sexual development at every cycle of red blood cell invasion [16]. Several studies have observed infected mosquitoes after feeding on blood containing gametocyte densities as low as 0.25–0.3 gametocytes/microlitre of blood, well below the threshold for detection by routine microscopy [7, 17–21]. As a consequence of the abundant presence of submicroscopic densities of gametocytes in clinical and asymptomatic infections [22–24], they represent a silent infectious reservoir in the population. In the last 20 years, sensitive molecular techniques based on sexual stage-specific mRNA transcripts have been developed to improve the detection and quantification of both gametocyte sexes. *Pfs25* mRNA has been widely used as a mature gametocyte marker [19–21, 25] and was recently confirmed to be female-specific or at least considerably female-biased [26, 27]. Based on RNA-seq analysis, *PfMGET* was recently presented as a novel male-specific gametocyte marker [26, 28]. The use of intron-spanning primers allows for sensitive detection of MG in samples of naturally infected parasite carriers [28]. Thus far a combination of separate *Pfs25* and *PfMGET* qRT-PCR assays has been used to estimate gametocyte sex ratios in natural and controlled infections [7, 15, 28–30]. However, estimating sex ratios by using two separate qRT-PCR assays may affect assay precision and throughput. Here, a novel target for FG is proposed, *CCp4*, that was previously identified as a gametocyte-specific transcript [31] and allows for intron-spanning primer design. This manuscript describes a one-step multiplex qRT-PCR assay for robust assessments of gametocyte sex ratios at densities below the microscopic threshold for gametocyte detection.

## Methods

### Selection of male and female marker transcript

The selection of the male marker *PfMGET* was described previously [28]. The female marker *CCp4* was identified by integrating transcriptomics data and validated as gametocyte-specific [31].

### Preparation of gametocyte material

Sex-sorted gametocytes were generated as described previously [26, 28]. In brief, cultures of the PfDynGFP/P47mCherry line [26] were treated with *N*-acetyl glucosamine and stage V gametocytes were FACS sorted for their fluorescence signal (MG are sorted as GFP-positive/mCherry-negative, FG as mCherry-positive/GFP-negative) and afterwards counted with a Bürker-Türk counting chamber. For both MG and FG tenfold dilution series were prepared in whole-blood in the range of 10^6^/mL to 10^1^/mL and stored in RNAProtect to serve as standard curves for gametocyte quantification.

### Preparation of ring stage parasites to assess transcript stage specificity

Asexual parasites of the NF54 strain were synchronized by the selection of late trophozoites and schizonts as described [28]. In brief, a 63% Percoll density gradient was followed by a 5% sorbitol treatment, killing the remaining schizonts after 5 h and ensuring tight synchronization. NF54 ring stage parasites were harvested 10–20 h after the Percoll treatment and stored in lysis buffer (5.25 M GuSCN; 50 mM Tris–HCl pH6.4; 20 mM EDTA; 1.3% Triton X-100) for later analysis.

To obtain pure asexual stage reference material without plausible contamination by gametocytes, ring stage parasites of the gametocyte-deficient F12 clone were used that have a loss-of-function mutation in the gene encoding the gametocyte master transcription factor AP2-G [32]. In addition, ring stage parasites were generated from the recently described AP2-G knock-down line 3D7/AP2-G-GFP-DDglmS that does not express AP2-G when grown in the presence of 2.5 mM (d)-+-glucosamine (GlcN) (Sigma Aldrich) [33]. Under these conditions both the *ap2*-*g*-*gfp*-*dd* transcript and AP2-G-GFP-DD protein are degraded, resulting in no gametocyte production. Parasites were synchronized twice 16 h apart to obtain an 8-h growth window. 30 mL parasite culture at 3–4% parasitaemia and 5% haematocrit was harvested at 8–16 h post invasion. Parasites were released from infected RBCs by saponin lysis and total RNA was directly isolated using Ribozol (Amresco) according to the manufacturer’s manual.

### Samples from naturally infected gametocyte carriers

Samples from two previously published clinical trials in light microscopy-positive gametocyte carriers from Kenya [28] and Mali [15] were used to directly compare gametocyte density estimates using the *PfMGET*/*CCp4* multiplex assay with the previous qRT-PCR assays targeting *PfMGET* (male) and *Pfs25* (female) transcripts separately. The multiplex assay was performed on Kenyan samples collected prior to treatment (day 0; n = 31), and after treatment on day 2 (n = 16), day 7 (n = 46), and day 14 with dihydroartemisinin–piperaquine (DP) alone or with primaquine (n = 28; total n = 121). The samples from Mali that were included in the current study were collected on day 7 after treatment with either DP (n = 15) or DP + methylene blue (15 mg/kg given daily for the first 3 days of treatment; DP + MB, n = 19) or sulfadoxine–pyrimethamine and amodiaquine (SP–AQ, n = 19) or SP–AQ with a single dose of primaquine (0.25 mg/kg given together with the first dose of SP–AQ, SP–AQ + PQ, n = 19) [15]. In both studies considerable variation in gametocyte densities and sex ratios was previously reported.

### Nucleic acid extraction and target amplification

Total nucleic acids were extracted from 50 µL whole blood in five volumes RNAProtect with the MagNAPure LC automated extractor (Roche) using the MagNAPure LC Total Nucleic Acid Isolation Kit—High Performance, with the exception of F12- and 3D7/AP2-G-GFP-DDglmS-derived material (bulk Ribozol (Amresco) extraction according to the manufacturer’s manual). Total nucleic acids were eluted in 50 µL of MagNAPure elution buffer, of which 5 µL was used in the multiplex assay. For the multiplex assay, we used the Luna^®^ Universal Probe One-Step RT-qPCR Kit (NEB). Gene IDs of the male and female markers and respective primer and probe sequences can be found in Table 1, for additional primer sequences see Additional file 1. Probe and primer concentrations were optimized (see Additional file 1) to obtain efficient amplification of both targets. The optimal conditions are summarized in Table 2. Negative controls were run to ensure there were no unspecific signals detected from no-template controls, or from total nucleic acids without reverse transcription (intron-spanning primers do not bind to genomic DNA); and melt curves were visually inspected.

**Table 1:** Primer and probe sequences for qRT-PCR assays, with references for earlier reported methods and primers

Gene ID Name	Fwd primer seq Rev primer seq	Probe seq	Fluoro-phore
PF3D7_1031000 *Pfs25* [36]	GAAATCCCGTTTCATACGCTTG AGTTTTAACAGGATTGCTTGTATCTAA	–	–
PF3D7_1031000 *Pfs25* [34]		6FAM-ccgtttcatacgcttgtaa-MGB	FAM
PF3D7_0903800 *CCp4* (MPX)	CACATGAATATGAGAATAAAATTG* TAGGCGAACATGTGGAAAG	AGCAACAACGGTATGTGCCTTAAAACG	Texas Red
PF3D7_0903800 *CCp4* (qRT-PCR)	CACATGAATATGAGAATAAAATTG* TAGGCGAACATGTGGAAAG	–	–
PF3D7_1469900 *PfMGET* (MPX)	CGGTCCAAATATAAAATCCTG* TGTGTAACGTATGATTCATTTTC	CAGCTCCAGCATTAAAAACAC	FAM
PF3D7_1469900 *PfMGET* (qRT-PCR, [28])	CGGTCCAAATATAAAATCCTG* GTGTTTTTAATGCTGGAGCTG	–	–

*MPX* multiplex assay, *qRT-PCR* quantitative real time reverse transcription-PCR

*Intron-spanning

**Table 2:** Multiplex conditions for male–female assay

	Concentration	Program	
Female primers CCp4	900 nM	55 °C 15 min	RT-step
Female probe—Texas Red	200 nM	95 °C 1 min	
Male primers PfMGET	225 nM	95 °C 10 s	45 cycles
Male probe—FAM	200 nM	60 °C 1 min	
Input total nucleic acid	5 µL		

Serial dilutions of all ring stage materials were used to detect ring stage transcripts (*SBP*-*1*) and early gametocyte transcripts (*Pfg27*, *Pfs16*, *Pfg14*-*744*, *Pfg14*-*748*) as well as the mature gametocyte transcripts *CCp4*, *PfMGET* and *Pfs25*. Total nucleic acids were used for the intron-containing genes *SBP*-*1*, *Pfg14*-*744*, *Pfg14*-*748*, *CCp4* and *PfMGET* while *Pfs16*, *Pfg27* and *Pfs25* required RQ1 DNase I treatment (Promega). cDNA was prepared with the High Capacity cDNA Reverse Transcription Kit (Applied BioSystems) and 2 μL of cDNA was run in the GoTaq qPCR Master Mix (Promega). All primers were used at 900 nM, except for *SBP1*, *PfMGET* and *Pfs25* which were run at 225 nM primers.

### Synthetic RNA standard curve material

Linear dsDNA templates for the target regions of *CCp4* and *PfMGET* were synthesized by BaseClear B.V. the Netherlands (for sequences, see Additional file 2) and purified by agarose gel electrophoresis. Bulk RNA was transcribed with the MEGAShortscript T7 high yield transcription kit (Invitrogen) at 10–50 nM dsDNA input according to the manufacturer’s instructions. Transcription samples were DNaseI treated with the TURBO DNA-*free* kit (Invitrogen) and subsequently purified over an RNeasy mini spin column (Qiagen). RNA standards were calibrated against sex-sorted gametocyte standards, both standards were prepared using tenfold serial dilutions. Copy numbers in initial samples were quantified on a Qubit 2 (ThermoFisher), absolute RNA amounts were calculated into copy numbers with the specific sequence weight.

### Gametocyte transcript stability testing

Synchronized mature gametocytes (NF54, 1.8% parasitaemia) were diluted in whole EDTA-blood starting at concentrations of 10^5^ gametocytes/mL. These samples were either stabilized by adding five volumes of RNAProtect (Qiagen) or left unstabilized (no protective buffer added) for further treatments: One to three aliquots were kept at room temperature (22–25 °C) for 0 h, 1 h, 2 h, 4 h, 6 h, 8 h or 24 h before freezing them at − 80 °C and subsequent processing. A subset of samples (n = 3) in RNAProtect (added at 0 h) were additionally freeze–thawed five times (37 °C/− 80 °C cycling for at least 1 h each) *before* extraction of nucleic acids. The stability of the transcripts *after* extraction was also tested by freeze–thaw cycles at which the samples were left at room temperature (22–25 °C) or 37 °C for 1 h, interspersed by at least 1 h at − 20 °C.

### Statistical analysis

Graphs and statistical analyses were made with GraphPad Prism (version 5.0.3) or R statistical software (version 3.4.0). The concordance between separate qRT-PCR assays and the multiplex approach was assessed by estimating the slope and 95% confidence interval (95% CI) in linear regression. No statistical comparisons were made on the CT values of different marker genes since this was beyond the scope of the current manuscript and meaningful comparisons would require a larger number of replicates. Where transcript abundance differences were estimated, this was based on the assumption of doubling of transcript after each cycle (1 Ct difference), regardless of reaction efficiencies.

## Results

### Male and female gametocyte transcripts are detected in low densities of gametocytes

**Fig. 1:**
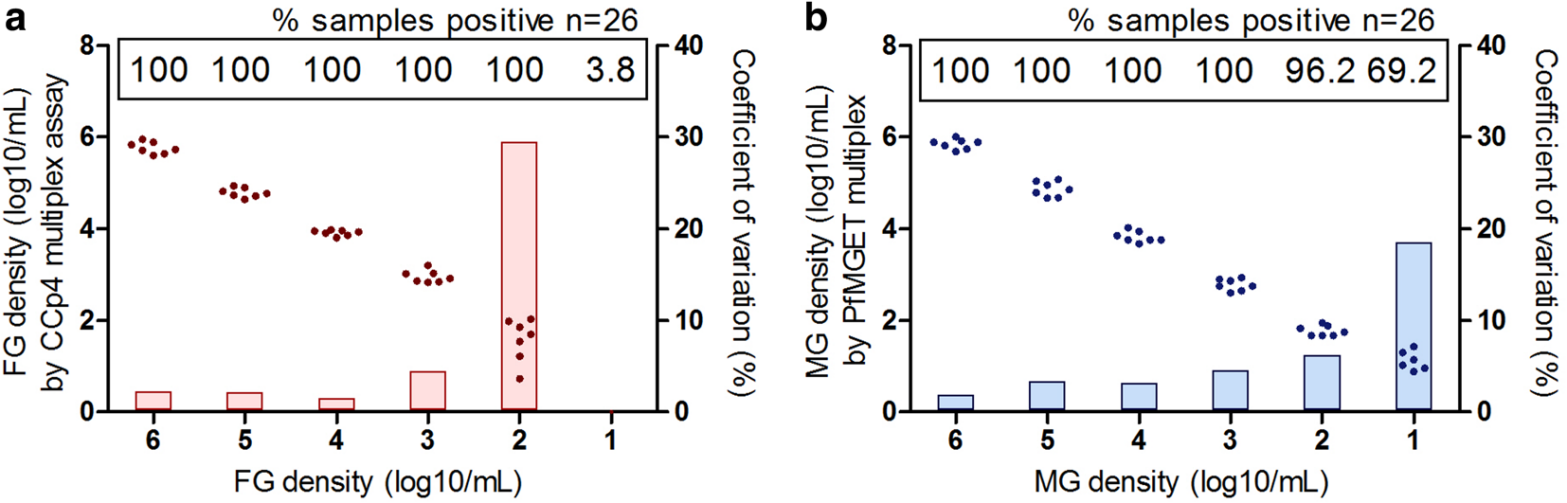
Limit of detection and variation of the gametocyte multiplex assay. Individual results of 8 technical replicates for female-sorted (**a**) and male-sorted (**b**) gametocytes of decreasing densities. Seven measurements (dots) obtained by calculating the reference from one randomly chosen dilution series. The coefficient of variation (filled bars) was calculated as standard deviation/mean of the respective calculated densities. Sample positivity in n = 26 independent experiments is indicated in the white boxes

A multiplex assay was developed to target the female- and male-specific gametocyte transcripts *CCp4* and *PfMGET* simultaneously by intron-spanning primers. Amplification from genomic DNA was absent when qRT-PCRs were performed without reverse transcription. Protocol optimization included limiting the primer concentration for the male target, which is explained in detail in Additional file 1 and resulted in an assay with two equally efficient amplification reactions for both target transcripts. Sorted MG and FG of the NF54-derived fluorescent reporter line *PfDyn*GFP/*PfP47*mCherry [26] were used in dilution series to determine the limit of detection (LOD), limit of quantification (LOQ) and variation in the multiplex assay. In an octuplicate run of female and male standard curve material, the coefficient of within-run variation was assessed (Fig. 1a, b), which is very low (2–3%) for both target transcripts at high gametocyte densities, but increasing to 6% or 29% at densities of 100 sex-sorted gametocytes/mL for *PfMGET* and *CCp4*, respectively. The LOD is 100 MG or FG/mL (0.1/µL) with 96.2% or 100% of all experiments at this density (n = 26) positive for *PfMGET* or *CCp4*, respectively. Even at 10 MG/mL (0.01 MG/µL), the male signal was detected in the majority of experiments (18 of 26, 69.2%), but with an increased variation coefficient (18%). The LOQ was hence equal to the LOD for MG (10^2^ MG/mL or 0.1 MG/µL) while for FG the LOD was 10^2^ FG/mL (0.1 FG/µL) and the LOQ was higher (10^3^ FG/mL or 1 FG/µL).

### Low densities of male and female gametocyte transcripts are detected in high densities of asexual parasites

**Fig. 2:**
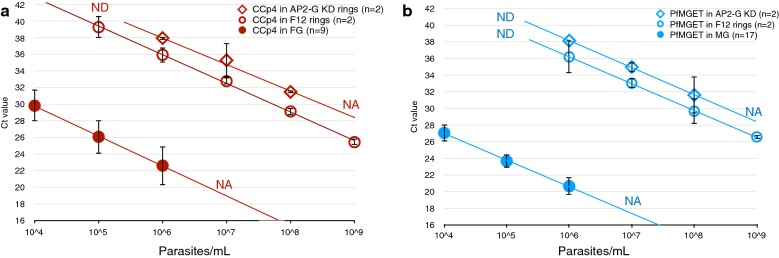
Stage-specificity of the multiplex assay. Female-specific (**a**) and male-specific (**b**) transcripts are detected in high concentrations of ring stage parasites of the F12 line and the 3D7/AP2-G-GFP-DDglmS line). Mean Ct values ± 2SD for varying numbers of independent experiments: n = 2 and n = 9 for *CCp4* in ring stage parasites and female gametocytes; n = 2 and n = 17 for *PfMGET* in ring stage parasites and male gametocytes, respectively. *NA* not available/not measured (samples at these concentrations were not available), *ND* not detected. Lines represent fitted linear regression curves

Whilst previous studies reported negligible *Pfs25* and male gametocyte transcripts in asexual (ring stage) parasites [25, 34, 35], low levels of *CCp4*, *Pfs25* and *PfMGET* transcripts were detected in pure asexual parasite material (Fig. 2). About 10,000 ring stage parasites from the gametocyte-deficient F12 *P. falciparum* line [32] provided a similar quantity of *CCp4* transcript as one FG and 100,000 F12 ring stages carried *PfMGET* transcript equivalent to one MG. In addition, ring stage parasites also contained *Pfs25* transcripts. Their concentration was about 100,000-fold less than FG (Additional file 1: Figure S2A). Interestingly, compared to F12 ring stages the expression of these gametocyte markers in ring stages of the 3D7/AP2-G-GFP-DDglmS line [33] was slightly lower when AP2-G was not expressed. Under these conditions, the fold-change in transcript between asexual rings and FG increased to 50,000-fold for *CCp4* and to 200,000- to 300,000-fold for *PfMGET* (MG over asexual rings). Ring stage material of the F12 line and 3D7/AP2-G-GFP-DDglmS was then compared with the gametocyte-producing NF54 line for the expression of markers that are transcribed from or at onset of sexual differentiation (*Pfg27*, *Pfs16*, *Pfg14*-*744* and *Pfg14*-*748*). The results indicate low-level contamination with early gametocytes in NF54 compared to both gametocyte-non producing lines. Consistently, all markers were expressed at higher levels in NF54 ring stages compared to F12 and 3D7/AP2-G-GFP-DDglmS (Additional file 1: Figure S3).

The measured background expression of *PfMGET* and *CCp4* transcripts in asexual ring stages leads to the following precautions, based on a conservative estimate of fold change of transcript levels (compared to F12 rings): for the detection of MG and FG, the presence of asexual parasites at densities above 1000 parasites/µL can result in a false-positive signal for FG whilst asexual parasites at densities above 10,000 parasites/µL can also result in a false-positive signal for MG (Table 3).

**Table 3:** The consequences of low level gametocyte transcripts in asexual parasites and gametocytes of the opposite sex for assessing gametocyte prevalence and density

Asexual parasite density		False positive signal in gametocyte quantification		Trust prevalence/estimated density if density is significantly above	
Log10/mL		FG	MG	FG	MG
4	10/µL	0.001/µL	0.0001/µL	Any	Any
5	100/µL	0.01/µL	0.001/µL	Any	Any
6	1000/µL	*0.1*/*µL*	0.01/µL	0.1/µL	Any
7	10,000/µL	*1*/*µL*	*0.1*/*µL*	1/µL	0.1/µL
8	100,000/µL	*10*/*µL*	*1*/*µL*	10/µL	1/µL

Numbers in italics are above the limit of detection. Other estimates are presented to illustrate the marginal impact of signal derived from asexual parasites on gametocyte quantification. The false positive signal strength for female (FG, *CCp4*) and male target (MG, *PfMGET*) is based on the comparison of NF54 (sorted) gametocytes and F12 ring stages

**Fig. 3:**
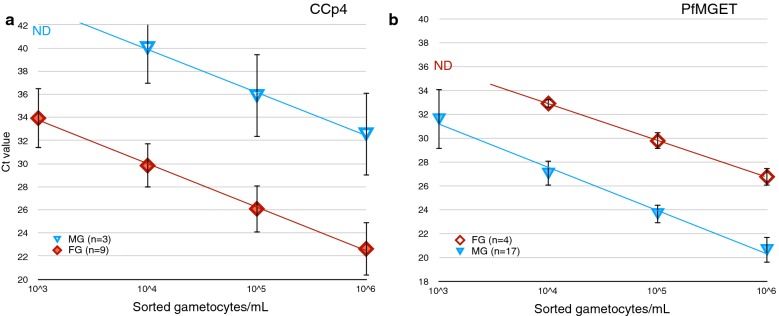
Sex-specificity of the multiplex assay. Female-specific (**a**) and male-specific (**b**) transcripts are detected in high concentrations of gametocytes of the opposite sex (empty symbols). Mean Ct values ± 2SD for varying numbers of independent experiments: n = 3 and n = 9 for *CCp4* in male and female gametocytes; n = 4 and n = 17 for *PfMGET* in female and male gametocytes, respectively. *ND* not detected. Lines represent fitted linear regression curves

Both *CCp4* and *PfMGET* were also moderately expressed in the opposite-sex gametocyte, detectable at concentrations of 10,000/mL or higher (Fig. 3). The opposite sex (MG) accounted for 0.1% (1 in 1000) false positive FG with *CCp4* (Fig. 3a), and about every 100 FG accounted for 1 MG (1% false positives) in *PfMGET*-based detection (Fig. 3b). *Pfs25* shows higher background than *CCp4* in the opposite sex: 0.5% (1 in 200, Additional file 1: Figure S2B).

### The male/female gametocyte multiplex assay corroborates earlier density estimates in naturally infected individuals

**Fig. 4:**
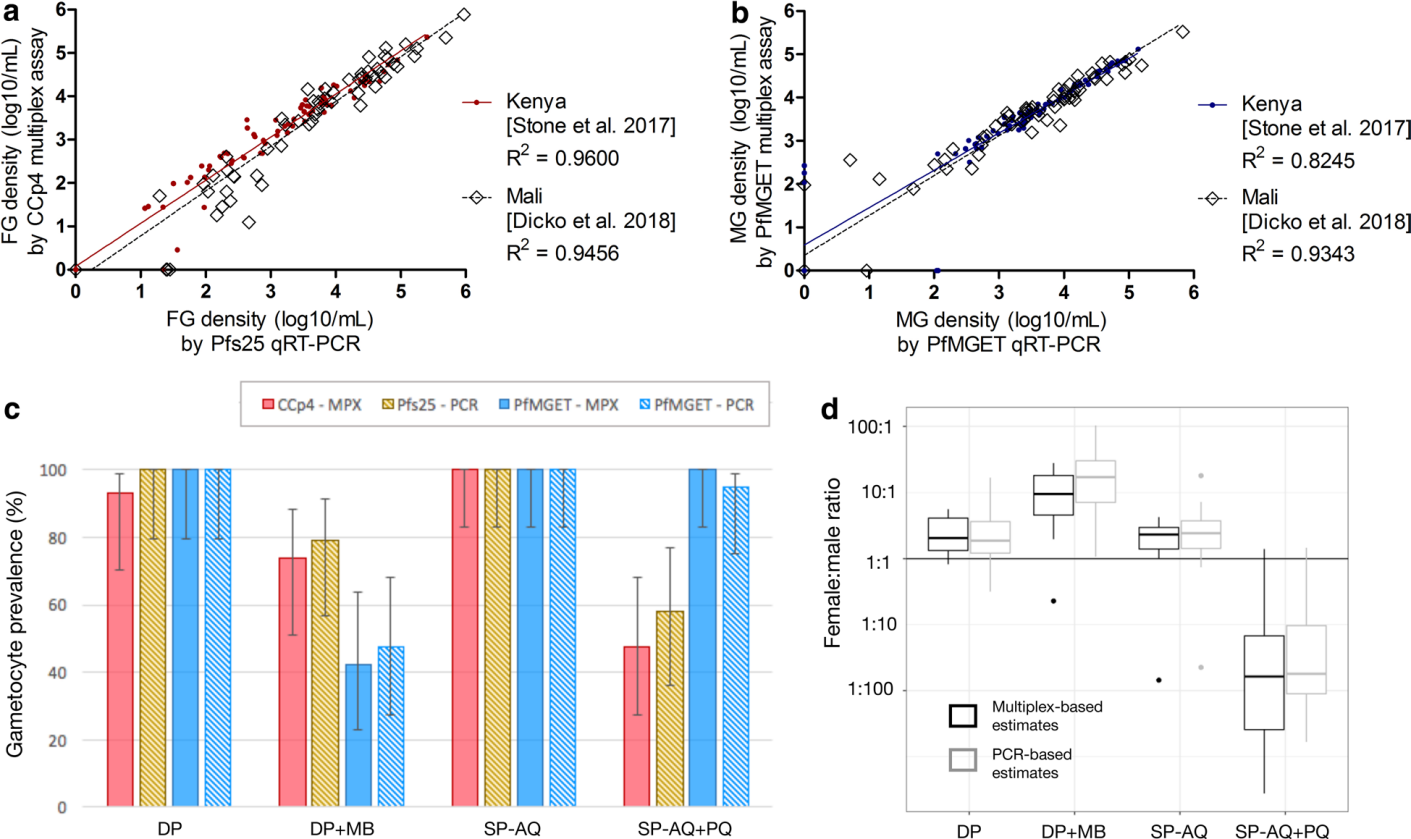
Performance of the multiplex-assay on clinical trial samples. **a**, **b** Agreement with previously measured gametocyte densities with single assays, using *Pfs25* (**a**) and *PfMGET* (**b**) transcripts. The linear regression was fitted as follows [95% confidence interval]: for FG y = 0.9944 [0.9497–1.040]x + 0.0751 [− 0.0627 to 0.2130] [28], y = 1.033 [0.977–1.092]x − 0.2554 [0.4619–0.04893] [15] and for MG y = 0.8626 [0.7744–0.9507]x + 0.5919 [0.2802–0.9037] [28], y = 0.9217 [0.8633–0.9800]x + 0.3508 [0.1515–0.5500] [15]. **c** Gametocyte prevalence as determined by single or multiplex qPCR for four treatment arms (Dicko et al. 2018), 7 days after treatment with either DP, dihydroartemisinin–piperaquine (n = 15); DP + MB, dihydroartemisinin–piperaquine + methylene blue (n = 19); SP–AQ, sulfadoxine–pyrimethamine and amodiaquine (n = 19) or SP–AQ and a single dose of primaquine (n = 19). **d** Sex ratios of gametocytes determined by multiplex or individual qPCR; samples as in **c**

Estimates of MG and FG are highly correlated with the estimates obtained by *Pfs25* qRT-PCR (FG for Kenya and Mali combined, R^2^ = 0.9473, p < 0.001) and *PfMGET* qRT-PCR (MG combined, R^2^ = 0.8869, p < 0.001) (Fig. 4a, b) using a selection of samples from clinical trials in Kenya (n = 84 samples) [28] and Mali (n = 72 samples) [15]. In the Malian study [15], extreme gametocyte sex ratios were reported and multiplex qRT-PCR-estimates for sex-specific gametocyte densities (Fig. 4a, b), prevalence (Fig. 4c) and sex ratios (Fig. 4d) were in agreement with the conventional quantification by qRT-PCR targeting *Pfs25* for FG [36] or *PfMGET* transcripts for MG [28].

A linear regression curve was fitted for single versus multiplex assay density estimates. For the MG quantification, there were indications that the slope deviated from 1 (95% CI 0.7744–0.9507 for the study in Kenya and 0.8633–0.9800 for the study in Mali) but resulting estimates in sex ratio were highly similar to those obtained after separate qRT-PCR (Fig. 4d).

### Male and female targets show similar stability under sub-optimal sample storage conditions

Differences in transcript stability upon delays in sample processing or following freeze–thaws may affect gametocyte quantification of field samples. To resemble freshly taken blood samples with delays in sample processing, whole blood samples with NF54 parasites containing a mix of male and female gametocytes were stored at room temperature (22–25 °C) without an RNA-protecting agent. Transcript abundance of *CCp4*, *PfMGET* and *Pfs25* was determined at baseline (0 h) and after 1 h, 2 h, 4 h, 6 h, 8 h or 24 h. All targets had stable Ct values after 1 h without RNA-protecting agent (n = 1). *PfMGET* and *CCp4* showed delayed Ct values after 2 h without protection (n = 2, Additional file 1: Figure S4). To study the effect of freezing/thawing after storage, blood was stored in RNAprotect and subjected to five freeze–thaw cycles at 37 °C prior to nucleic acid extraction. A minimal transcript loss was observed (0.523 and 0.245 Ct difference to baseline for *PfMGET* and *CCp4*, respectively, Additional file 1: Table S1). Extracted total nucleic acids were more stable and showed no loss in signal (0.08 and 0.02 Ct reduction) compared to baseline for *PfMGET* and *CCp4*, after five freeze–thaw cycles at room temperature for the sorted trend line material (Additional file 1: Table S1).

### Synthetic RNA standards can be used to estimate gametocyte densities and copy numbers per gametocyte

In vitro-synthesized RNA of the target regions of *CCp4* and *PfMGET* was used in serial dilutions alongside sorted gametocyte standards (see Additional file 1: Figure S5A, B). The synthetic RNA standards were used to estimate the copy numbers in the gametocyte reference material (see Additional file 1: Figure S5C–E). For *CCp4*, estimated transcript copies were 4 (95% CI 3.2–5.1) mRNA copies per female gametocyte and for *PfMGET* 9.8 (95% CI 8.9–10.2) copies per male gametocyte. For comparison, female gametocytes had an average of 231.7 (95% CI 199.1–269.8) Pfs25 copies per gametocyte.

## Discussion

In the current study, a multiplex assay for the rapid quantification of female and male gametocytes is reported. The assay utilizes a new female gametocyte marker *CCp4* in conjunction with the reported male gametocyte marker *PfMGET* [28]. The use of intron-spanning primers allows simultaneous quantification of male and female-specific transcript levels in total nucleic acids without prior DNase I treatment. The presented analysis concludes low but non-negligible gametocyte transcripts in gametocytes of the opposite sex and gametocyte-free ring-stage asexual parasites. The stability of *CCp4* and *PfMGET* transcripts was similar under suboptimal storage conditions; gametocytes can be reliably detected and quantified at densities 0.1–1 gametocyte/μL.

CCp4 is a member of the LCCL-domain containing adhesion protein family and orthologous to LAP6 in *Plasmodium berghei*, where this gene is reported to be translationally repressed with protein expression occurring at the ookinete stage only [37]. Earlier, a DOZI (development of zygote inhibited) knock out indicated that *LAP6* transcripts (then called PB000955.03.0) are accumulated in an mRNA storage complex [38]. In *P. falciparum*, the CCp4 protein is predominantly expressed at the gametocyte stage, with only minor expression in male gametocytes [39] and no evidence for translational repression. Gametocyte-specific *CCp4* transcripts were reported in an integrated analysis of eight *Plasmodium* transcriptomes [31]. The initial validation in qRT-PCR experiments confirmed *CCp4* expression to be at least 1000-fold upregulated in gametocytes of different *P. falciparum* strains compared to asexual blood stages. The 1000-fold higher mRNA levels in FG over MG reported here confirm and exceed the previous estimates by RNAseq (38-fold higher in females by RPKM values) [26].

Unlike the commonly used female marker *Pfs25*, *CCp4* allows the design of intron-spanning primers. The current assay utilizes intron-spanning primers of both male and female reporter genes with two marker-specific probes. Importantly, the multiplex gametocyte assay can be performed on total nucleic acids without DNase I treatment, which may affect gametocyte detection at low densities [28, 36]. The multiplex male–female assay is thus faster than separate assays. Sensitivity for detecting female gametocyte was lower than for the single qRT-PCR targeting *Pfs25*, which is at least in part explained by a lower estimated number of *CCp4* transcripts per female gametocyte as compared to *Pfs25*. The LOD for female and male gametocytes in the multiplex assay is 0.1/μL, well below the limit of microscopic detection and in the same range as other molecular sex-specific assays [28, 34, 35]. More sensitive total gametocyte assays have been reported [40] but the current multiplex LOD allows for the detection of infections that are likely to be transmissible to mosquitoes. An increasing likelihood of mosquito infections is consistently observed at gametocyte densities above 1–5 gametocyte per microlitre [7, 29, 41]. The current assay reliably quantifies gametocytes at these densities. The lower sensitivity to detect female gametocytes as compared to *Pfs25* may be a concern in studies where very low overall gametocyte densities are observed [29, 30] but the operational attractiveness of a multiplex assay that does not require DNase treatment is considerable for many other studies.

Previous work indicated that the stability of transcripts is a relevant concern when estimating gametocyte prevalence or density [36, 41]. When assessing gametocyte sex ratio, transcript stability is a particular concern since differences in the stability between target transcripts may affect bias estimates. In a limited set of experiments there were no indications for differences in the stability of *PfMGET* and *CCp4* transcripts, provided samples are transferred to RNA-protective buffer within 1–2 h of blood collection. Freeze–thaw cycles resulted in limited RNA loss once blood samples are in this protective buffer. A similar stability of male or female signal is of particular relevance for studies conducted in low resource settings where there may be challenges in ensuring optimal storage conditions. Repeated or prolonged freeze–thaw cycles may thus affect overall gametocyte detection or quantification [41, 42] but current results indicate they would not disproportionally affect MG or FG quantification and thus sex-ratio estimates.

The presented multiplex assay is a fast route to accurate *P. falciparum* sex ratio determination, saving about 25% of the time—with similar material costs—compared to two separate assays of which one requires a DNase treatment step. Medium sample through-put in 96-well format is the recommended application, providing accurate gametocyte quantification and sex ratio determination for blood and culture samples.

Previous studies concluded no or negligible *Pfs25* transcript numbers in asexual parasites. In the current set of experiments, we detected gametocyte transcripts in different preparations of ring-stage asexual parasite material. Whilst low-level contamination of gametocytes in supposedly pure asexual parasites may have contributed to the detection of *CCp4*, *Pfs25* and *PfMGET* transcripts in asexual parasite material from the NF54 strain, the detection of these transcripts in the gametocyte-deficient F12 line and under knock-down conditions for AP2-G in a more recent gametocyte-less line 3D7/AP2-G-GFP-DDglmS [33] provides convincing evidence for low-level expression of *Pfs25*, *CCp4* and *PfMGET* in asexual ring stage parasites. The current findings of detectable transcript expression for all gametocyte markers in high densities of asexual blood stages despite different strategies to avoid gametocyte contamination have implications for past and future gametocytaemia estimates. Whilst the difference in transcript abundance between gametocytes and asexual parasites is sufficiently pronounced to conclude a marginal impact on gametocyte density estimates, gametocyte prevalence estimates may be inflated in some populations. Given the high parasitaemia of some acute malaria infections (with densities typically above 10,000 parasites/µL [43] as opposed to asymptomatic infections where densities commonly lie below 10 parasites/µL [44]), earlier studies recruiting clinical malaria cases may have overestimated gametocyte prevalence by molecular assays. In studies with asymptomatic parasite carriers and in low-endemic settings, where lower asexual parasite densities dominate, this overestimation will be less pronounced and often negligible. A previously reported rapid decline in gametocyte prevalence based on *Pf25* mRNA detection in the first 3 days following treatment of high-density asexual infections [45] may thus be (partially) explained by the detection of *Pfs25* transcripts arising from asexual parasites, whilst the gradual decline in gametocyte prevalence following treatment of lower-density asymptomatic asexual parasite carriers [15, 46] or gametocyte transcript kinetics in the period following asexual parasite clearance may better reflect gametocyte clearance and gametocyte half-life [47]. With a better appreciation of caveats in gametocyte detection, the molecular tools for gametocyte detection are of value for studies aiming to quantify the human infectious reservoir for malaria, the kinetics of gametocyte production and the impact of interventions on gametocyte carriage. As a consequence of the detection of low level transcripts of gametocyte markers in rings, it is advised to report gametocyte prevalence in samples with parasite densities > 1000 parasites/µL together with a qualifying remark on the reliability of gametocyte prevalence and quantification. The presented results suggest that gametocyte prevalence determined in samples below assay-specific cut-off values indeed can be trusted (Table 3). Stating the limitations of molecularly determined gametocyte prevalence for densities, if required, will re-confirm the validity of molecular gametocyte detection.

With a cautious interpretation of low gametocyte density estimates in samples with high concurrent asexual parasite densities, molecular gametocyte diagnostics such as the multiplex assay presented in this manuscript are valuable tools to obtain sensitive and robust estimates of gametocyte prevalence and density. With these tools, gametocyte densities and sex ratios can be assessed across the gametocyte density range that is likely to contribute to onward transmission to mosquitoes [7, 19], which in many settings is well below the threshold for detection by microscopy.

## Conclusion

The presented multiplex qPCR assay is a valuable addition to gametocyte diagnostic tools. A new female gametocyte marker gene, *CCp4* was introduced and benchmarked against *Pfs25* transcript-based quantification. The use of *CCp4* and *PfMGET* as targets has the following advantages: throughput is facilitated by the use of intron-spanning primers which allow amplification of mRNA only without a DNA digestion step, sensitivity is sufficiently high to detect and quantify all potentially transmitting gametocyte densities. The target mRNAs result in a similar detectability of male and female gametocytes and show similar stability under suboptimal storage conditions, allowing robust gametocyte sex-ratio estimates in field studies.

## Additional files

**Additional file 1.** Optimization of the multiplex amplification assay and different gametocyte targets.

**Additional file 2.** Protocol for multiplex amplification assay.

## References

[1] WHO (2017). World malaria report 2017.

[2] Ranson H, Lissenden N (2016). Insecticide resistance in African Anopheles mosquitoes: a worsening situation that needs urgent action to maintain malaria control. Trends Parasitol.

[3] Dondorp AM, Nosten F, Yi P, Das D, Phyo AP, Tarning J (2009). Artemisinin resistance in *Plasmodium falciparum* malaria. N Engl J Med.

[4] Churcher TS, Dawes EJ, Sinden RE, Christophides GK, Koella JC, Basáñez M-G (2010). Population biology of malaria within the mosquito: density-dependent processes and potential implications for transmission-blocking interventions. Malar J.

[5] Robert V, Read AF, Essong J, Tchuinkam T, Mulder B, Verhave JP (1996). Effect of gametocyte sex ratio on infectivity of *Plasmodium falciparum* to *Anopheles gambiae*. Trans R Soc Trop Med Hyg.

[6] Mitri C, Thiery I, Bourgouin C, Paul REL (2009). Density-dependent impact of the human malaria parasite *Plasmodium falciparum* gametocyte sex ratio on mosquito infection rates. Proc Biol Sci.

[7] Bradley J, Stone W, Da DF, Morlais I, Dicko A, Cohuet A (2018). Predicting the likelihood and intensity of mosquito infection from sex specific *Plasmodium falciparum* gametocyte density. Elife.

[8] Sinden RE, Strong K (1978). An ultrastructural study of the sporogonic development of *Plasmodium falciparum* in *Anopheles gambiae*. Trans R Soc Trop Med Hyg.

[9] Talman AM, Domarle O, McKenzie FE, Ariey F, Robert V (2004). Gametocytogenesis: the puberty of *Plasmodium falciparum*. Malar J.

[10] Robert V, Macintyre K, Keating J, Trape J-F, Duchemin J-B, Warren M (2003). Malaria transmission in urban sub-Saharan Africa. Am J Trop Med Hyg.

[11] Gbotosho GO, Sowunmi A, Happi CT, Okuboyejo TM (2011). *Plasmodium falciparum* gametocyte carriage, sex ratios and asexual parasite rates in Nigerian children before and after a treatment protocol policy change instituting the use of artemisinin-based combination therapies. Mem Inst Oswaldo Cruz.

[12] Reece SE, Drew DR, Gardner A (2008). Sex ratio adjustment and kin discrimination in malaria parasites. Nature.

[13] Paul RE, Raibaud A, Brey PT (1999). Sex ratio adjustment in *Plasmodium gallinaceum*. Parassitologia.

[14] Delves MJ, Ruecker A, Straschil U, Lelièvre J, Marques S, López-Barragán MJ (2013). Male and female *Plasmodium falciparum* mature gametocytes show different responses to antimalarial drugs. Antimicrob Agents Chemother.

[15] Dicko A, Roh ME, Diawara H, Mahamar A, Soumare HM, Lanke K (2018). Efficacy and safety of primaquine and methylene blue for prevention of *Plasmodium falciparum* transmission in Mali: a phase 2, single-blind, randomised controlled trial. Lancet Infect Dis.

[16] Sinden RE (1983). The cell biology of sexual development in Plasmodium. Parasitology.

[17] Githeko AK, Brandling-Bennett AD, Beier M, Atieli F, Owaga M, Collins FH (1992). The reservoir of *Plasmodium falciparum* malaria in a holoendemic area of Western Kenya. Trans R Soc Trop Med Hyg.

[18] Boudin C, Olivier M, Molez JF, Chiron JP, Ambroise-Thomas P (1993). High human malarial infectivity to laboratory-bred *Anopheles gambiae* in a village in Burkina Faso. Am J Trop Med Hyg.

[19] Schneider P, Bousema JT, Gouagna LC, Otieno S, van de Vegte-Bolmer M, Omar SA (2007). Submicroscopic *Plasmodium falciparum* gametocyte densities frequently result in mosquito infection. Am J Trop Med Hyg.

[20] Ouédraogo AL, Bousema T, Schneider P, de Vlas SJ, Ilboudo-Sanogo E, Cuzin-Ouattara N (2009). Substantial contribution of submicroscopical *Plasmodium falciparum* gametocyte carriage to the infectious reservoir in an area of seasonal transmission. PLoS ONE.

[21] Ouédraogo AL, Gonçalves BP, Gnémé A, Wenger EA, Guelbeogo MW, Ouédraogo A (2016). Dynamics of the human infectious reservoir for malaria determined by mosquito feeding assays and ultrasensitive malaria diagnosis in Burkina Faso. J Infect Dis.

[22] Babiker HA, Abdel-Wahab A, Ahmed S, Suleiman S, Ranford-Cartwright L, Carter R (1999). Detection of low level *Plasmodium falciparum* gametocytes using reverse transcriptase polymerase chain reaction. Mol Biochem Parasitol.

[23] Nassir E, Abdel-Muhsin A-MA, Suliaman S, Kenyon F, Kheir A, Geha H (2005). Impact of genetic complexity on longevity and gametocytogenesis of *Plasmodium falciparum* during the dry and transmission-free season of eastern Sudan. Int J Parasitol.

[24] Schneider P, Bousema T, Omar S, Gouagna L, Sawa P, Schallig H (2006). (Sub)microscopic *Plasmodium falciparum* gametocytaemia in Kenyan children after treatment with sulphadoxine–pyrimethamine monotherapy or in combination with artesunate. Int J Parasitol.

[25] Schneider P, Schoone G, Schallig H, Verhage D, Telgt D, Eling W (2004). Quantification of *Plasmodium falciparum* gametocytes in differential stages of development by quantitative nucleic acid sequence-based amplification. Mol Biochem Parasitol.

[26] Lasonder E, Rijpma SR, van Schaijk BCL, Hoeijmakers WAM, Kensche PR, Gresnigt MS (2016). Integrated transcriptomic and proteomic analyses of *P. falciparum* gametocytes: molecular insight into sex-specific processes and translational repression. Nucleic Acids Res.

[27] Walzer KA, Kubicki DM, Tang X, Chi J-TA (2018). Single-cell analysis reveals distinct gene expression and heterogeneity in male and female Plasmodium falciparum gametocytes. mSphere.

[28] Stone W, Sawa P, Lanke K, Rijpma S, Oriango R, Nyaurah M (2017). A Molecular assay to quantify male and female *Plasmodium falciparum* gametocytes: results from 2 randomized controlled trials using primaquine for gametocyte clearance. J Infect Dis.

[29] Collins KA, Wang CYT, Adams M, Mitchell H, Rampton M, Elliott S (2018). A controlled human malaria infection model enabling evaluation of transmission-blocking interventions. J Clin Invest.

[30] Reuling IJ, van de Schans LA, Coffeng LE, Lanke K, Meerstein-Kessel L, Graumans W (2018). A randomized feasibility trial comparing four antimalarial drug regimens to induce *Plasmodium falciparum* gametocytemia in the controlled human malaria infection model. Elife.

[31] Meerstein-Kessel L, van der Lee R, Stone W, Lanke K, Baker DA, Alano P (2018). Probabilistic data integration identifies reliable gametocyte-specific proteins and transcripts in malaria parasites. Sci Rep.

[32] Alano P, Roca L, Smith D, Read D, Carter R, Day K (1995). *Plasmodium falciparum*: parasites defective in early stages of gametocytogenesis. Exp Parasitol.

[33] Filarsky M, Fraschka SA, Niederwieser I, Brancucci NMB, Carrington E, Carrió E (2018). GDV1 induces sexual commitment of malaria parasites by antagonizing HP1-dependent gene silencing. Science.

[34] Schneider P, Reece SE, Van Schaijk BCL, Bousema T, Lanke KHW, Meaden CSJ (2015). Quantification of female and male *Plasmodium falciparum* gametocytes by reverse transcriptase quantitative PCR. Mol Biochem Parasitol.

[35] Santolamazza F, Avellino P, Siciliano G, Yao FA, Lombardo F, Ouédraogo JB (2017). Detection of *Plasmodium falciparum* male and female gametocytes and determination of parasite sex ratio in human endemic populations by novel, cheap and robust RTqPCR assays. Malar J.

[36] Wampfler R, Mwingira F, Javati S, Robinson L, Betuela I, Siba P (2013). Strategies for detection of Plasmodium species gametocytes. PLoS ONE.

[37] Saeed S, Carter V, Tremp AZ, Dessens JT (2013). Translational repression controls temporal expression of the *Plasmodium berghei* LCCL protein complex. Mol Biochem Parasitol.

[38] Mair GR, Braks JAM, Garver LS, Wiegant JCAG, Hall N, Dirks RW (2006). Regulation of sexual development of plasmodium by translational repression. Science.

[39] Scholz SM, Simon N, Lavazec C, Dude MA, Templeton TJ, Pradel G (2008). PfCCp proteins of *Plasmodium falciparum*: gametocyte-specific expression and role in complement-mediated inhibition of exflagellation. Int J Parasitol.

[40] Essuman E, Grabias B, Verma N, Chorazeczewski JK, Tripathi AK, Mlambo G (2017). A novel gametocyte biomarker for superior molecular detection of the *Plasmodium falciparum* infectious reservoirs. J Infect Dis.

[41] Gonçalves BP, Kapulu MC, Sawa P, Guelbéogo WM, Tiono AB, Grignard L (2017). Examining the human infectious reservoir for *Plasmodium falciparum* malaria in areas of differing transmission intensity. Nat Commun.

[42] Pett H, Gonçalves BP, Dicko A, Nébié I, Tiono AB, Lanke K (2016). Comparison of molecular quantification of *Plasmodium falciparum* gametocytes by Pfs25 qRT-PCR and QT-NASBA in relation to mosquito infectivity. Malar J.

[43] Slater HC, Ross A, Ouédraogo AL, White LJ, Nguon C, Walker PGT (2015). Assessing the impact of next-generation rapid diagnostic tests on *Plasmodium falciparum* malaria elimination strategies. Nature.

[44] Imwong M, Stepniewska K, Tripura R, Peto TJ, Lwin KM, Vihokhern B (2016). Numerical distributions of parasite densities during asymptomatic malaria. J Infect Dis.

[45] Sawa P, Shekalaghe SA, Drakeley CJ, Sutherland CJ, Mweresa CK, Baidjoe AY (2013). Malaria transmission after artemether–lumefantrine and dihydroartemisinin–piperaquine: a randomized trial. J Infect Dis.

[46] Dicko A, Brown JM, Diawara H, Baber I, Mahamar A, Soumare HM (2016). Primaquine to reduce transmission of *Plasmodium falciparum* malaria in Mali: a single-blind, dose-ranging, adaptive randomised phase 2 trial. Lancet Infect Dis.

[47] Bousema T, Okell L, Shekalaghe S, Griffin JT, Omar S, Sawa P (2010). Revisiting the circulation time of *Plasmodium falciparum* gametocytes: molecular detection methods to estimate the duration of gametocyte carriage and the effect of gametocytocidal drugs. Malar J.

