# Supplementary material for: A multiplex assay for the sensitive detection and quantification of male and female Plasmodium falciparum gametocytes
Source: Malar J. 2018 Nov 29;17:441. doi: 10.1186/s12936-018-2584-y (PMC6267050; doi:10.1186/s12936-018-2584-y)
Supplement: Supplementary file 1 — Additional file 1. Optimization of the multiplex amplification assay and different gametocyte targets. [file 12936_2018_2584_MOESM1_ESM.docx]

Additional file 1

A multiplex assay for the sensitive detection and quantification of male and female *P. falciparum* gametocytes

Lisette Meerstein-Kessel^1,2^, Chiara Andolina^1^, Elvira Carrio^3,4^, Almahamoudou Mahamar^5^, Patrick Sawa^6^, Halimatou Diawara^5^, Marga van de Vegte-Bolmer^1^, Will Stone^7^, Katharine A. Collins^1^, Petra Schneider^8^, Alassane Dicko^5^, Chris Drakeley^7^, Ingrid Felger^3^, Till Voss^3^*^,^*^4^ , Kjerstin Lanke^1^*, Teun Bousema^1^*

Corresponding author: Teun Bousema

1 Department of Medical Microbiology, Radboud university medical center, Nijmegen, The Netherlands

2 Centre for Molecular and Biomolecular Informatics, Radboud Institute for Molecular Life Sciences, Radboud university medical center, Nijmegen, The Netherlands

3 Swiss Tropical and Public Health Institute, Basel, Switzerland

4 University of Basel, Basel, Switzerland

5 Malaria Research and Training Centre, University of Science, Techniques and Technologies of Bamako, Bamako, Mali

6 ﻿Human Health Division, International Centre for Insect Physiology and Ecology, Mbita Point, Kenya

7 Department of Immunology and Infection, London School of Hygiene and Tropical Medicine, Faculty of Infectious and Tropical Diseases, London, United Kingdom

8 Institute of Evolutionary Biology and Institute of Immunology and Infection Research, School of Biological Sciences, University of Edinburgh, Edinburgh, United Kingdom.

**Optimization of the multiplex amplification assay**

To achieve the best possible sensitivity of the assay, input volumes of total extracted nucleic acids were varied between 1ul, 2ul, 3ul, 4ul and 5 ul. With 5ul input, the lowest concentrations of gametocytes were detected most frequently. In high excess of male transcripts, a flattened curve for *CCp4* amplification was observed. To recover efficiency for the *CCp4* reaction, the primer concentration for the *PfMGET* transcript was lowered from 900nM to 225nM (Figure S1). The opposite effect for *PfMGET* on female gametocyte material was never observed, the *PfMGET* reaction efficiency was consistently high. The efficiency averaged at 103.1% for *PfMGET*; at 92.47% for *CCp4*.


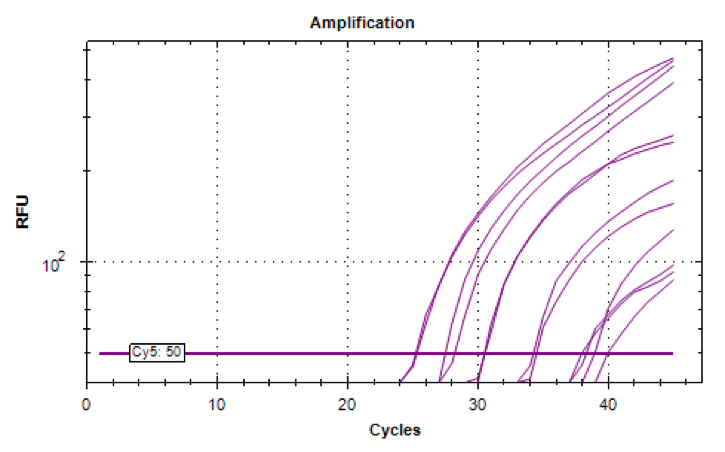

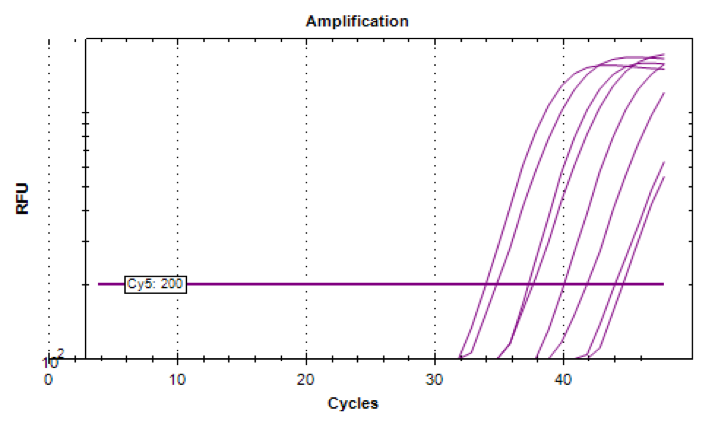


**B**

**A**

Figure S1

**Female signal in male sorted gametocytes**. Representative amplification curves of CCp4 transcript at **A** equal primer concentrations (900nM each) for CCp4 and PfMGET, **B** male-limited primer concentrations (CCp4 900nM, PfMGET 225 nM). Serial dilutions of male gametocytes (starting at 10^6/mL) run in duplicate.


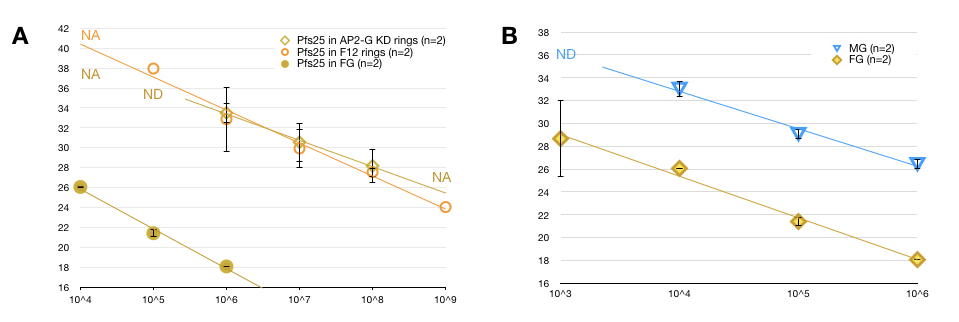


Figure S2

**Stage- and sex-specificity of Pfs25 in qRT-PCR.**

Pfs25 transcripts are detected in high concentrations of ring stage parasites of the F12 line or of the 3D7/AP2-G-GFP-DDglmS line (AP2-G KD rings) (A) and in male gametocytes (B). Lines are linear regression curves. ND, not detectable, NA, sample not available.


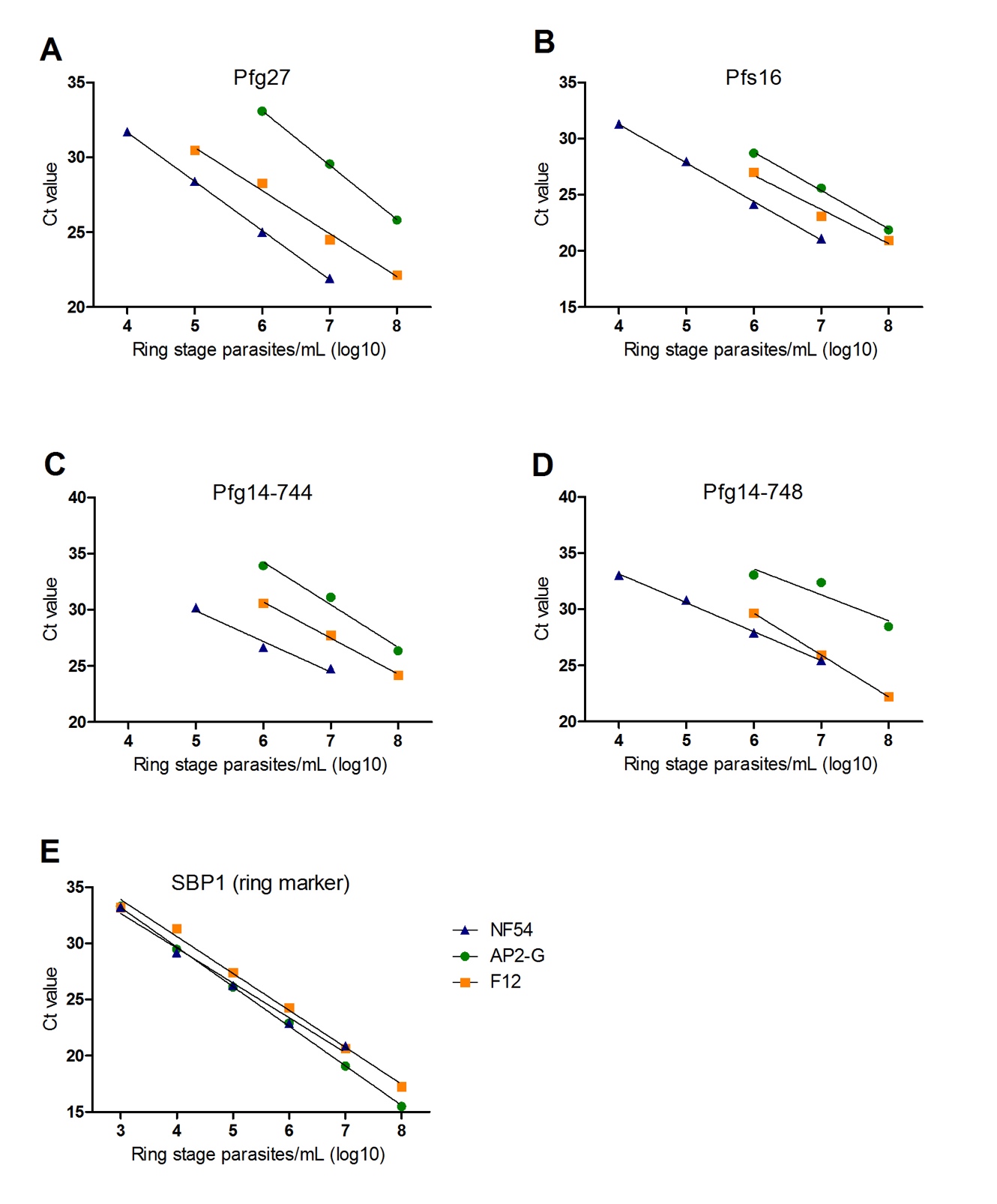


Figure S3

**qRT-PCR (GoTaq) for early gametocyte markers (A-D) and ring stage marker (E) on serial dilutions of ring stage material (A+B DNase I treated, C-E reverse transcription from total nucleic acids)**

Highest NF54 ring concentration available was 10^7/mL. AP2-G rings (green) isolated from the 3D7/AP2-G-GFP-DDglmS line under AP2-G knock down conditions, F12 rings (orange) and NF54 rings (blue). Transcript abundance of **A** Pfg27 (PF3D7_1302100), forward primer (fwd) CTTAGCAAGGATCCTGAGAAGTTT, reverse primer (rev) GTTGACAATGTTATCTTGGACACGT (Brancucci et al. 2014); **B** Pfs16 (PF3D7_0406200), fwd AGTTCTTCAGGTGCCTCTCTTCA , rev AGCTAGCTGAGTTTCTAAAGGCA (Brancucci et al. 2014); **C** Pfg14-744 (PF3D7_1477300), fwd ATTCATTATGTATAATGGCTCTG (intron-spanning), rev CAGAAGCATAATTTGAAAAAG C, **D** Pfg14-748 (PF3D7_1477700), fwd CTGAATTTTGTGTTATGGTTATTC (intron-spanning), rev AAGCATCATCATCATTGTAGTTC; **E** SBP1 (PF3D7_0501300) fwd GCAAAACAAGCCGTACATGTTG, rev TTGCTAGGTAATATCCTTTTCTTTTTCC (intron-spanning) (Tadesse et al. 2017)

All n=1 for NF54 and F12, n=2 for AP2-G rings. Lines are linear regression curves.


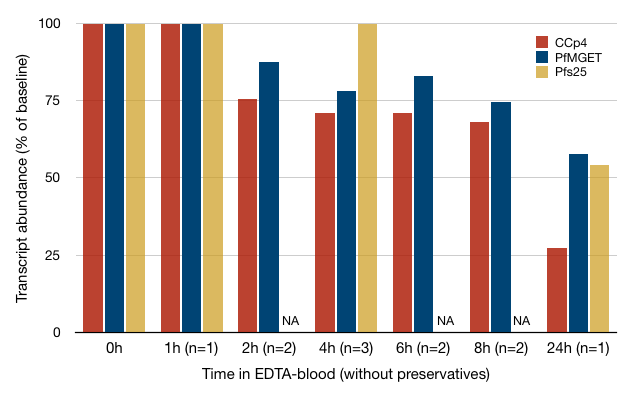


Figure S4

**Stability of gametocyte transcripts without RNA-protecting agents.**

Transcripts of *CCp4*, *PfMGET* and *Pfs25* were measured in blood that was stored at room temperature without RNA-protecting agents. Two independent experiments included storage of one or two biological replicates with storage for 0h (t0), 1h, 2h, 4h, 6h, 8h and 24h. Transcript input was calculated assuming doubling of transcript numbers at an increase of one Ct-value during qPCR. NA, not available (*Pfs25* was only included in one set of experiments, n=1 for all conditions)

**
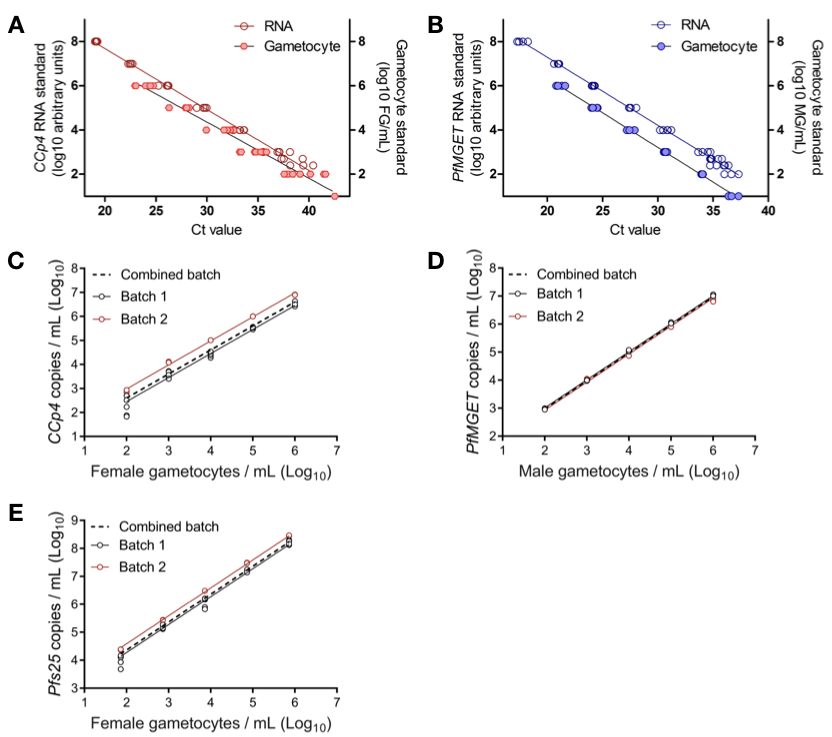
**

**Figure S5**

**In vitro RNA standards as reference material and quantification of transcripts per gametocyte.** **A-B** Slopes are equal when transcripts are quantified from sex-sorted gametocytes (filled circles) or synthetic RNA standards (open circles) for *CCp4* (A) and *PfMGET* (B), all conditions n=5. **C-E** Fitted linear regression for two batches of sex-sorted gametocytes and calculated copy numbers for *CCp4* (C), *PfMGET* (D) and Pfs25 (E). *CCp4* transcript copies varied from 3.2-5.1 mRNA copies per female gametocyte; *PfMGET* transcripts varied from 8.9-10.2 copies per male gametocyte and Pfs25 transcripts from 199.1-269.8 copies per female gametocyte (all ranges given as 95% CI). Initial linear regression analysis was performed for each marker and the slopes were not different from 1: for *CCp4* 1.036 (95%CI 0.962-1.11), *PfMGET* 0.9967 (95% CI 0.9756-1.018) and *Pfs25* 1.038 (95% CI 0.9925-1.084). For the calculation of the conversion factor (copy numbers per gametocyte), linear regression was performed a second time with the slope constrained to 1.

**Table S1: Ct losses compared to baseline (t0, no freeze-thaw), averaged over 2-4 concentrations ranging from 1,000 to 100,000 gametocytes/mL**. Freeze-thawing of blood in five volumes of RNAProtect (RNAP, Qiagen) involved cycling the material between -80C and 37 C for at least one hour each. Freeze-thawing of extracted nucleic acids involved >1 hour each at -20C or room temperature (22-25C). NA, nucleic acids

|  | 5 freeze-thaws of whole blood+RNAP at 37C (average loss in Ct compared to t0) ± 2SD | 5 freeze-thaws of extracted NA at RT (average loss in Ct compared to t0) ± 2SD |
| --- | --- | --- |
| **CCP4** | 0.245±0.071 | 0.02±0.026 |
| **PfMGET** | 0.523±0.583 | 0.08±0.142 |
| **Pfs25** | 0.297±1.028 | 0.14±0.190 |

**References**

Brancucci, N.M.B. et al., 2014. Heterochromatin Protein 1 Secures Survival and Transmission of Malaria Parasites. *Cell Host & Microbe*, 16(2), pp.165–176.

Tadesse, F.G. et al., 2017. Molecular Markers for Sensitive Detection of Plasmodium falciparum Asexual Stage Parasites and their Application in a Malaria Clinical Trial. *The American Journal of Tropical Medicine and Hygiene*, 97(1), pp.188–198.
