# Supplementary material for: A multiplex assay for the sensitive detection and quantification of male and female Plasmodium falciparum gametocytes
Source: Malar J. 2018 Nov 29;17:441. doi: 10.1186/s12936-018-2584-y (PMC6267050; doi:10.1186/s12936-018-2584-y)
Supplement: Supplementary file 2 — Additional file 2. Protocol for multiplex amplification assay. [file 12936_2018_2584_MOESM2_ESM.docx]

ADDITIONAL PROTOCOL

A multiplex assay for the sensitive detection and quantification of male and female *P. falciparum* gametocytes (Meerstein-Kessel et al.)

**PfMGET-CCp4 qRT-PCR for male-female gametocyte quantification**

> Starting material is (MagNaPure) extracted Total NA (usually in hard-shell 96 wells plates), use filtertips and set-up reactions in PCR cabinet.

> Prepare plate layout in Excell for your qRT-PCR plate.

qRT-PCR with the Luna Universal Probe OneStep RT-qPCR kit (NEB, order at Bioke):

> Thaw buffer and primers on ice

> qPCR: 15 ul of mixture + 5 ul of Total NA

>> Prepare the mastermix according to the scheme below, always prepare +6 reactions extra. Thaw Luna reaction mix on ice, keep Luna RT enzyme mix on ice and start preparing mixture immediately. First mix Luna Reaction mix by pipetting up and down:

ddH2O 3.47 ul

Female CCp4 primers (50 uM) 0.36 ul (900 nM)

Female CCp4 TexasRed-probe (100 uM) 0.04 ul (200 nM)

Male PfMGET primers (50 uM) 0.09 ul (225 nM)

Male PfMGET FAM-probe (100 uM) 0.04 ul (200 nM)

Luna Reaction mix 2x 10 ul

Luna RT enzyme mix 20x 1 ul .

Template Total NA 5 ul

Total reaction volume 20 ul

> Fill out 15 ul/well with repetitive pipet and 0.5 ml combitip in PCR cabinet. Use the BioRad hard-shell PCR 96 well plates (HSP9635)

> Add 5 ul of Total NA with multichannel and seal with plastic seal

> Spin 20 sec in plate spinner

> Run PCR program: 55°C 15 min > 95°C 1 min > 95°C 10 sec, 60°C 1 min, 44 cycles

Data analysis:

> After the run select fluorophore FAM (PfMGET Male signal), and deselect the others > OK

> Fill in your standard curve dilutions

> Settings > Baseline settings > Apply fluorescence drift correction and log scale

> Set threshold: Single threshold, set to 200 (or applicable value to reduce background noise

> Save graphs and results to program of choice

> select fluorophore Texas Red (CCp4 Female signal), and deselect the others > OK

> Fill in your standard curve dilutions

> Settings > Baseline settings > Apply fluorescence drift correction and log scale

> Save graphs and results to program of choice

**Primers/Probes:**

PfMGET Forward primer 5’- cggtccaaatataaaatcctg -3’ (DST Sigma)

PfMGET Reverse primer 5’- tgtgtaacgtatgattcattttc-3’(DST Sigma)

PfMGET Probe: 5’-FAM-cagctccagcattaaaaacac-BHQ1-3’ (HPLC BioLegio)

CCp4 Forward primer 5’- cacatgaatatgagaataaaattg-3’ (DST Sigma)

CCp4 Reverse primer 5’- taggcgaacatgtggaaag-3’(DST Sigma)

CCp4 Probe: 5’-TexasRed-agcaacaacggtatgtgccttaaaacg-BHQ2-3’ (HPLC BioLegio)

For the use of synthetic RNA standards:

Synthetic dsDNA templates were custom-ordered with the following sequences:

*CCp4*

GGGGGAAAATtaatacgactcactata*gggagattgtcgagcaagcttcaaagaatatttatctaataagaaaatatttttaaaatataatggtacctattgtttataccctgaaaatgttatagaaaatgatataattgaaatagccaccgatgaatgtgataaagtagcacatgaatatgagaataaaattgtttggttttcgttcgataatggaagattaagaagcaacaacggtatgtgccttaaaacgtacaaaaatattttagtgctttccacatgttcgcctaataaaaatgaaaagtcagaaatgtggaagatagatgaagaaacacatttaaaaaatgaaaacaacaattgtgctcaaatagctgggaataaattattttcttttacatgcaataatttatcaactaaagaatttgaacaatccatattttcttcagacgaattatatttaatgaaaacaagatattc*

477 bp dsDNA ; *450 nt ssRNA*

*PfMGET*

GGGGGAAAATtaatacgactcactata*gggagaatgagccgcataaaggacaaaagcacaacactttcaaaaaataaaaaattcggtccaaatataaaatcctgttcagaagaaaaaaaaagtatcctggtaaaaaacagctccagcattaaaaacacaaaaaagggatttttaattgatgaagataatataattaacaaaataaaagagctaaatataattctgttacaaaataaagacgaaatagataatctgaagcaagaaaatgaatcatacgttacacaaataaccaattttatgaataagtgcaaagaactacaaaatatatgtaacgataaagatattatacttttcaaatataaacaagaagaaaagaatcttttacaattaatagattcttataaaaaagaaaaggaagaattacaaaatgaaatagaaaaactagaaaataacatccaatctacaaaaggacaattgttggtacaaaataatgaaatagatatattaaagaaagaaataaaagaaaa*

527 bp dsDNA; *500 nt ssRNA*

In green: T7 promotor sequence

In yellow: qPCR amplicon

In italic: RNA transcript

Underlined: qPCR primers

- Gel-purify the dsDNA templates and measure concentration (Nanodrop)
- RNA transcription with the MEGAshortscript T7 transcription kit (Invitrogen) with 10-50 nM dsDNA template according to the manufacturer’s instructions
- DNaseI treatment with TURBO DNAfree-kit (Invitrogen)
- RNeasy spin-column purification (Qiagen)
- If available: Measure RNA concentration with Qubit RNA HS kit on Qubit Fluorometer (LifeTechnologies)
- Calculate copy numbers and prepare dilution series in MP extracted negative blood
- Otherwise: Adjust copy numbers/RNA input to a known sex-sorted gametocyte standard
- Run a calibration plate with both standards
